# Women engaged in prostitution and COVID-19: why are they excluded from socially vulnerable groups?

**DOI:** 10.11606/s1518-8787.2022056004229

**Published:** 2022-02-23

**Authors:** Michelle Ishida Chiang, Maitê Leite Basile, Ana Beatriz Pereira de Souza, Isabella Dastler Moccagatta, Julia Rabello Guerra Vieira, Thaís Rocha Lourenço, Mariana Langanke Gonçalves, Helena Afférri Fernandes Pinto, Rosane Lowenthal, Michele Lacerda Pereira Ferrer, Giselle Burlamaqui Klautau

**Affiliations:** I Faculdade de Ciências Médicas Santa Casa de São Paulo São Paulo SP Brasil Faculdade de Ciências Médicas da Santa Casa de São Paulo. Curso de Graduação em Medicina. São Paulo,SP, Brasil; II Faculdade de Ciências Médicas Santa Casa de São Paulo Departamento de Saúde Mental São Paulo SP Brasil Faculdade de Ciências Médicas da Santa Casa de São Paulo. Departamento de Saúde Mental. São Paulo,SP, Brasil; III Faculdade de Ciências Médicas Santa Casa de São Paulo Departamento de Atenção Primária à Saúde São Paulo SP Brasil Faculdade de Ciências Médicas da Santa Casa de São Paulo. Departamento de Atenção Primária à Saúde. São Paulo,SP, Brasil; IV Faculdade de Ciências Médicas Santa Casa de São Paulo Departamento de Clínica Médica São Paulo SP Brasil Faculdade de Ciências Médicas da Santa Casa de São Paulo. Departamento de Clínica Médica. São Paulo,SP, Brasil

**Keywords:** Women, Sex Workers, COVID-19, epidemiology, Risk Factors, Gender Inequality, Race Factors

## Abstract

This study analyzed the exposure of women engaged in prostitution in downtown São Paulo to COVID-19. This cross-sectional study had a convenience sample selected in May 2021. We interviewed 219, mostly black, middle-aged, poor women with comorbidities. Among them, 61 had shown COVID-19 symptoms, 23, tested positive for the disease, seven underwent hospitalization, and four reported post-COVID-19 complications. Only 26 (30.2%) had been vaccinated. In addition to gender, race, and class inequalities, these women suffer both from a higher risk of contracting COVID-19 due to their working conditions and from the subsequent worsening of that disease due to age and lack of vaccination.

## INTRODUCTION

The new coronavirus, SARS-CoV-2, causes COVID-19, an infectious disease^a^. As of July 4, 2021, Brazil has had approximately 18.6 million cases of the disease and 518,000 deaths^b^. In the state of São Paulo, vaccination began in January 2021, following priority criteria; first vaccinating healthcare providers, older adults, and persons with comorbidities^c^. In addition to these and according to social determinants of health, this vaccination plan also included highly socially vulnerable populations, but fail to include persons engaged in prostitution under the same criteria.

Despite severe socioeconomic inequalities already affecting the persons in the group of vulnerable populations, the COVID-19 pandemic caused a health, economic, political, and social crisis which further worsened these persons’ vulnerability^1^. Thus, women engaged in prostitution’s sociodemographic characteristics, health, access to services, exposure and history of contamination should inform the attention and care allocated toward this population, so as to include them in vaccination priority groups. We conducted this study to assess the exposure of women engaged in prostitution working in downtown São Paulo to COVID-19 and evaluate what preventive measures these women adopted against the disease.

## METHODS

An observational, cross-sectional study was conducted with data collected from a convenience sample between May 27 and 29, 2021, during an educational action organized by a non-governmental organization, the collective *Mulheres da Luz*, in partnership with the discipline of Infectious Diseases of the *Santa Casa de São Paulo* School of Medical Sciences. Women engaged in prostitution who attended the educational action, which took place at a square near the Luz subway station in downtown São Paulo, were included in our study. Informed consent forms were signed by all participants before research began. Interviews were conducted individually and outdoors, respecting social distancing protocols. This study was approved by the Research Ethics Committee of the *Irmandade da Santa Casa de Misericórdia* CAAE: 47175821.3.0000.5479.

Questions on sociodemographic data, work and behavior during the pandemic, prevention and exposure factors to SARS-CoV-2, comorbidities, medication, close contact with someone infected during the transmission period, whether they showed symptoms or tested positive for COVID-19, hospitalization, post-COVID-19 complications, and vaccination status were included in our questionnaire. At the time data were gathered, persons over 45 years of age with comorbidities or disabilities, healthcare providers over 30 years of age, puerperal and pregnant women, municipal transit workers, and homeless people over 18 years of age registered in reception centers were eligible for vaccination against COVID-19 in the municipality of São Paulo^d^.

Descriptive statistical analyses were described by means and standard deviations, and categorical variables, by absolute and relative frequencies.

## RESULTS

We evaluated a total of 219 women with a mean age of 41.3 years (SD = 12), a minimum age of 19 years, and a maximum age of 73 years. Of the total number of interviewees, 78.8% declared themselves to be cisgender, 69.3%, black or mixed, 42.7% live in rented homes, and 9.2% were homeless or in shelters. Regarding working habits, 34.2% reported working seven days a week, turning a weekly average of 18.5 tricks (SD = 14.7), with most women turning between 11 and 30 tricks per week (51.1%). Table 1 provides additional details.

**Table:** Sociodemographic characterization, and habits and behaviors of women engaged in prostitution in São Paulo during the COVID-19 pandemic, 2021 (n = 219).

	n	%
Age group		
19–30	51	23.3
31–40	59	26.9
41–50	60	27.4
51–60	33	15.1
≥ 61	16	7.3
Gender identity		
Cisgender	164	78.8
Transgender	44	21.1
Ethnicity		
White	64	29.4
Black	61	28.0
Mixed	90	41.3
Indigenous	3	1.4
Income group^a^		
A	0	0
B1	3	1.4
B2	7	3.2
C	77	35.2
D/E	132	60.3
Housing		
Streets or shelters	20	9.2
Pension	29	13.3
Provisionally with friends or family	14	6.4
Owner-occupancy	47	21.6
Rented house or apartment	93	42.7
Other	15	6.9
Weekly work frequency		
1	10	4.6
2	23	10.5
3	34	15.5
4	25	11.4
5	29	13.2
6	23	10.5
7	75	34.2
Number of tricks turned/week		
Minimum = 1 | Maximum = 112	Média = 18.5	DP = 14.7
Up to 10	74	33.8
11–20	62	28.3
21–30	50	22.8
31–40	14	6.4
More than 40	19	8.7
Tricks/day		
1	16	7.3
2	54	24.7
3	54	24.7
4	39	17.8
5	24	11.0
≥ 6	32	14.6
Trick location		
Hotel/motel	212	96.8
Car	21	9.6
Client’s house	17	7.8
Street	14	6.4
2In their own home	9	4.1
Other	4	1.8
Use of mask during sessions		
Yes	129	59.4
No	58	26.7
Occasionally	30	13.8
Symptoms of COVID-19 infection (n = 61)		
Fever	34	55.7
Dry cough	38	62.3
Fatigue	28	45.9
Nasal discharge	16	26.2
Anosmia	21	34.4
Ageusia	18	29.5
Diarrhea	14	23.0
Nausea or vomiting	10	16.4
Other	4	6.6
Positive result for COVID-19 (n = 197)		
Yes	23	11.7
No	174	88.3
Hospitalization for the disease (n = 156)		
Yes	7	4.5
No	149	95.5
COVID-19 complications (n = 23)		
Yes	4	17.4%
No	19	82.6%

^a^ According to the 2013 Brazilian Economic Classification Criteria (CCEB 2013) of the Brazilian Association of Research Companies (ABEP).

In total, 166 women (77.2%) reported using masks while on the streets as a protective measure, whereas 27 failed to do so (12.6%). In our sample, 192 women (88.9%) reported wearing cloth masks, whereas 37 (17.1%) chose surgical, and seven, PFF2 ones (3.2%).

Of the 219 women interviewed, 23 (11.7%) reported having tested positive for COVID-19, of which seven had undergone hospitalization, and four, some complication due to the disease. Of the total number of participants in our study, 180 (82.6%) reported not having yet been vaccinated. Among those who already met vaccination criteria at the time of the interview, 60 (69.8%) lacked immunization.

## DISCUSSION

Since the beginning of the COVID-19 pandemic, women engaged in prostitution have been unable to interrupt face-to-face services to conform to sanitary measures^1^.

Moreover, the atypical circumstances of the pandemic induce perverse effects, imposing greater risks on the most invisible sectors of society^2^. In this context, due to immunization plans excluding them from priority vulnerable groups and several national government plans, from financial aid, economic crises affect even more the already socioeconomically marginalized women engaged in prostitution. Moreover, even with the end of social isolation, the economic crisis due to the pandemic will persist, possibly leading to a greater number of persons engaged in prostitution, resulting in elevated levels of vulnerability^3^.

Our study found that comorbidities, and the lack of access to vaccination and to protective measures among these women are aggravating factors for the risk of infection by SARS-CoV-2, corroborating the data described in the recent literature^4^.

Among the 219 interviewees, 86 reported comorbidities, the most common of which were hypertension (AH) (40.7%), severe chronic pneumopathy (24.4%), immunosuppression (22.1%), and diabetes mellitus (19.8%). According to the 2020 Brazilian Guideline on Arterial Hypertension, 21.4% of the Brazilian population reports suffering from AH, a prevalence that increases to 65% in persons over 60 years of age^5^. In this study, however, interviewees’ mean age was 41.3 years, and only 7.3% of them were over 60 years of age. This age disparity allows us to infer that such women show an increased incidence of AH at earlier ages, possibly due to the exacerbation of other risk factors.

According to the National Immunization Plan (PNI) in Brazil, individuals with comorbidities belong to one of the vaccination priority groups against COVID-19. However, in this study, only 26 of the 86 women who reported having comorbidities had received immunization (30.2%). Thus, even if government agencies prioritize the ill, it seems that this information fails to reach the most vulnerable populations. Vaccination of women engaged in prostitution depends on their access to social and health centers^1^. The stigma hanging over them may be one of the factors distancing them from public social and health services, including immunization campaigns. Historically, society has viewed these women, and still does, as “disease reservoirs” transmitting STIs, disregarding the risk of illness they run.

Regarding masks, we found that most women used them (59.4%), but this proportion falls short of satisfactory due to the sanitary situation of COVID-19 and the mode of transmission of the virus. One explanation for this is that the number of their usual clients decreased due to the pandemic, which led these women to accept new clients in whom they had no trust or whose care against COVID-19 they could not know^6^. Moreover, these clients may demand the removal of their masks during sessions^6^. Thus, as with the fight against HIV, the fight against COVID-19 is extremely complex^6^ and, despite educational measures and donations of masks to these women, many continue to turn tricks without using them due to their financial needs. Thus, the difficulty in wearing a mask during sessions makes explicit the need for specific care for this population, such as vaccination priority.

## CONCLUSION

This study shows that the women engaged in prostitution interviewed were mostly black, middle-aged, poor, and unvaccinated against COVID-19. Thus, in addition to gender, race, and class inequalities, these women suffer both from a higher risk of contracting COVID-19 due to their working conditions and the subsequent worsening of that disease due to age and lack of vaccination. Other aspects, such as the prevalence of comorbidities, absence of the use of masks during sessions, and the impossibility of social distancing contribute to these factors, making the scenario even more delicate.

Finally, we must not underestimate the impact of the lack of access to information and to already available health services due to the notable precariousness of care for this population.

The multiple vulnerabilities shown, therefore, point to the pressing need for specific public policies for this population located in a vulnerable area such as downtown São Paulo. Currently, these women are invisible to society and to the Unified Health System (SUS) due to the lack of respect for its principles of universality, integrality, and equity of access to health. Thus, the visibility of women engaged in prostitution in all contexts, especially during the pandemic, is crucial for Brazilian public health to realize its principles.

## Footnotes

a Brazilian Ministry of Health, Health Surveillance Secretariat, Department of Immunization and Communicable Diseases, General Coordination of the National Immunization Program. National Plan for the Operationalization of the Vaccine against COVID-19. 4th ed. Brasília, DF; February 15, 2021. [cited 2021 Sep 12]. Available from: https://www.gov.br/saude/pt-br/media/pdf/2021/janeiro/29/PlanoVacinaoCovid_ed4_15fev21_cgpni_18h05.pdf

b Brazil: WHO Coronavirus Disease (COVID-19) dashboard with vaccination data. Geneva (CH): WHO; 2021 [cited 2021 Mar 12]. Available from: https://covid19.who.int/region/amro/country/br

c São Paulo (State). #Vacina Já. São Paulo; 2021 [cited 2021 Sep 12]. Available from: https://www.vacinaja.sp.gov.br/

d São Paulo Municipal Health Department. Instruction no. 21 for Dose Prioritization. Campaign Vaccinate Sampa against COVID-19. São Paulo; 2021 [cited 2021 Sep 18]. Available from: https://www.prefeitura.sp.gov.br/cidade/secretarias/saude/vigilancia_em_saude/doencas_e_agravos/coronavirus/index.php?p=312200
