# Supplementary material for: Women engaged in prostitution and COVID-19: why are they excluded from socially vulnerable groups?
Source: Rev Saude Publica. 2022 Feb 23;56:3. doi: 10.11606/s1518-8787.2022056004229 (PMC8868671; doi:10.11606/s1518-8787.2022056004229)
Supplement: Translation

# Translation: Mulheres em situação de prostituição e covid-19: por que excluídas dos grupos vulneráveis?

**Authors:** Michelle Ishida Chiang, Maitê Leite Basile, Ana Beatriz Pereira de Souza, Isabella Dastler Moccagatta, Julia Rabello Guerra Vieira, Thaís Rocha Lourenço, Mariana Langanke Gonçalves, Helena Afférri Fernandes Pinto, Rosane Lowenthal, Michele Lacerda Pereira Ferrer, Giselle Burlamaqui Klautau

**Affiliations:** I Brasil Faculdade de Ciências Médicas da Santa Casa de São Paulo. Curso de Graduação em Medicina. São Paulo, SP, Brasil; II Brasil Faculdade de Ciências Médicas da Santa Casa de São Paulo. Departamento de Saúde Mental. São Paulo, SP, Brasil; III Brasil Faculdade de Ciências Médicas da Santa Casa de São Paulo. Departamento de Atenção Primária à Saúde. São Paulo, SP, Brasil; IV Brasil Faculdade de Ciências Médicas da Santa Casa de São Paulo. Departamento de Clínica Médica. São Paulo, SP, Brasil

**Keywords:** Mulheres, Profissionais do Sexo, COVID-19, epidemiologia, Fatores de Risco, Iniquidade de Gênero, Fatores Raciais

## Abstract

Esta pesquisa analisou a exposição de mulheres em situação de prostituição no centro de São Paulo à covid-19. Este estudo transversal contou com amostra de conveniência selecionada em maio de 2021. Entrevistou-se 219 mulheres majoritariamente negras, de meia idade, pobres e com comorbidades. Dentre essas mulheres, 61 tiveram sintomas de covid-19, 23 com teste positivo,7 foram internadas e 4 relataram complicações pós-covid-19. Somente 26 (30,2%) haviam sido vacinadas. Além das desigualdades de gênero, raça e classe, essas mulheres são expostas a um maior risco de contraírem covid-19, devido às condições de trabalho e por apresentarem doença grave relacionada à idade e falta de vacinação.

## INTRODUÇÃO

A covid-19 é uma doença infecciosa causada pelo novo coronavírus, o SARS-CoV-2^a^. O Brasil foi afetado com aproximadamente 18,6 milhões de casos e 518 mil mortes até o dia 4 de julho de 2021^b^. Em São Paulo, deu-se início à vacinação em janeiro de 2021, seguindo critérios de prioridade, sendo primeiramente vacinados os profissionais da saúde, idosos e pessoas com comorbidades^c^. Além desses grupos prioritários, incluiu-se populações com elevada vulnerabilidade social, seguindo os critérios baseados nos determinantes sociais da saúde (DSS). As pessoas em situação de prostituição não foram inseridas em grupos prioritários por vulnerabilidade social.

No grupo das populações vulneráveis, que já representavam pessoas acometidas por desigualdades socioeconômicas severas, observou-se um agravamento ainda maior de suas vulnerabilidades em decorrência da crise sanitária, econômica, política e social durante a pandemia da covid-19^1^. Sob esse olhar, as mulheres em situação de prostituição deveriam receber atenção e cuidado direcionado para as suas demandas e vulnerabilidades a partir do conhecimento das características sociodemográficas, de saúde e acesso a serviços, exposição e histórico de contaminação, para que possam ser inseridas entre os grupos prioritários de vacinação. Este estudo foi realizado para compreender a exposição à covid-19 das mulheres em situação de prostituição no centro de São Paulo e identificar medidas preventivas adotadas contra a doença por estas mulheres.

## MÉTODOS

Foi realizado um estudo observacional, transversal, com coleta de dados em uma amostra de conveniência entre os dias 27 e 29 de maio de 2021, durante uma ação educativa organizada por uma Organização não-governamental, o coletivo Mulheres da Luz, em parceria com a disciplina de Infectologia da Faculdade de Ciências Médicas da Santa Casa de São Paulo. A pesquisa envolveu mulheres em situação de prostituição que compareceram ao local da ação, uma praça próxima ao metrô da Luz. Todas as participantes assinaram o termo de consentimento livre e esclarecido antes da aplicação da pesquisa. A entrevista foi realizada individualmente, ao ar-livre, respeitando os protocolos de distanciamento social. A pesquisa foi aprovada pelo Comitê de Ética em Pesquisa da Irmandade da Santa Casa de Misericórdia CAAE: 47175821.3.0000.5479.

O questionário de pesquisa incluiu dados sociodemográficos, informações sobre o trabalho e o comportamento durante a pandemia, fatores de prevenção e exposição ao SARS-CoV-2; além de comorbidades, uso de medicamentos, contato próximo com alguém infectado durante o período de transmissão, se apresentou sintomas ou testou positivo para covid-19, internação, complicações pós-covid-19 e vacinação. À época da coleta de dados, eram elegíveis para vacinação contra covid-19 no município de São Paulo pessoas com comorbidades e deficiências acima de 45 anos, profissionais de saúde acima de 30 anos, puérperas e gestantes, trabalhadores de transporte coletivo municipal, pessoas em situação de rua cadastradas em centros de acolhida acima de 18 anos^d^.

As análises estatísticas descritivas são apresentadas por meio de médias e desvios-padrões. As variáveis categóricas são descritas por meio de frequências absolutas e relativas.

## RESULTADOS

Foram avaliadas 219 mulheres com idade média de 41,3 anos (DP = 12,0), sendo a idade mínima de 19 anos e a máxima de 73 anos. Do total de entrevistadas, 78,8% se declararam cisgênero, 69,3% pretas e pardas, 42,7% moram em domicílio alugado e 9,2% em situação de rua ou abrigo. Em relação aos hábitos durante a situação de prostituição, 34,2% relataram fazer programas sete vezes na semana, sendo a média de 18,5 programas realizados por semana (DP = 14,7), com a maioria das mulheres realizando entre 11 e 30 programas por semana (51,1%). Dados adicionais podem ser encontrados na Tabela.

**Tabela:** Caracterização sociodemográfica, hábitos e comportamentos durante a pandemia de covid-19 em mulheres situação de prostituição em São Paulo, 2021 (n = 219).

	n	%
Faixa etária		
19–30	51	23,3
31–40	59	26,9
41–50	60	27,4
51–60	33	15,1
≥ 61	16	7,3
Identidade de gênero		
Cisgênero	164	78,8
Transgênero	44	21,1
Raça/cor		
Branca	64	29,4
Preta	61	28,0
Parda	90	41,3
Indígena	3	1,4
Classe de renda^a^		
A	0	0
B1	3	1,4
B2	7	3,2
C	77	35,2
D/E	132	60,3
Situação de moradia		
2Em situação de rua ou abrigo	20	9,2
Em pensão	29	13,3
Mora provisoriamente com amigos ou família	14	6,4
Casa própria	47	21,6
Casa ou apartamento alugado	93	42,7
Outros	15	6,9
Quantas vezes na semana faz o programa		
1	10	4,6
2	23	10,5
3	34	15,5
4	25	11,4
5	29	13,2
6	23	10,5
7	75	34,2
Quantidade de programas/semana		
Mínimo = 1 | Máximo = 112	Média = 18,5	DP = 14,7
até 10	74	33,8
11–20	62	28,3
21–30	50	22,8
31–40	14	6,4
mais de 40	19	8,7
Quantidade de programa/dia		
1	16	7,3
2	54	24,7
3	54	24,7
4	39	17,8
5	24	11,0
≥ 6	32	14,6
Local do programa		
Hotel/motel	212	96,8
Carro	21	9,6
Casa do cliente	17	7,8
Rua	14	6,4
Na própria moradia	9	4,1
Outros	4	1,8
Uso de máscara durante o programa		
Sim	129	59,4
Não	58	26,7
Às vezes	30	13,8
Sintomas por infecção de covid-19 (n = 61)		
Febre	34	55,7
Tosse seca	38	62,3
Fadiga	28	45,9
Coriza	16	26,2
Anosmia	21	34,4
Ageusia	18	29,5
Diarreia	14	23,0
Náusea ou vômito	10	16,4
Outro	4	6,6
Resultado positivo para covid-19 (n = 197)		
Sim	23	11,7
Não	174	88,3
Internação por conta da doença (n = 156)		
Sim	7	4,5
Não	149	95,5
Complicação da covid-19 (n = 23)		
Sim	4	17,4%
Não	19	82,6%

^a^ De acordo com o Critério de Classificação Econômica Brasil (CCEB 2013) da Associação Brasileira de Empresas de Pesquisa (ABEP).

O uso de máscaras na rua foi apontado como medida de proteção por 166 mulheres (77,2%) e 27 afirmaram não usar máscara (12,6%), a maioria delas (88,9%, 192 mulheres) afirmou utilizar máscaras de pano, a máscara cirúrgica foi escolhida por 37 dessas mulheres (17,1%) e sete delas optaram pelo modelo PFF2 (3,2%).

Das 219 mulheres entrevistadas, 23 (11,7%) relataram um resultado positivo para covid-19, sendo que sete afirmaram terem sido internadas e quatro tiveram alguma complicação. Do total participante da pesquisa, 180 (82,6%) afirmaram ainda não terem se vacinado. Dentre as que já tinham critérios para vacinação na data da entrevista, 60 (69,8%) não tinham ainda se imunizado.

## DISCUSSÃO

Desde o início da pandemia de covid-19, mulheres em situação de prostituição foram incapazes de interromper serviços presenciais para seguir medidas sanitárias^1^.

Ademais, as circunstâncias atípicas trazidas pelo advento da pandemia induzem efeitos perversos, impondo maiores riscos aos setores mais invisibilizados da sociedade^2^. Nesse contexto, as mulheres em situação de prostituição, que já eram socioeconomicamente marginalizadas, são ainda mais afetadas no período de crise econômica, pois além de serem excluídas dos grupos vulneráveis prioritários no plano de imunização, são também excluídas dos planos governamentais de auxílio financeiro em diversos países. Além disso, mesmo com o fim do isolamento social, a crise econômica persistirá, possivelmente levando a um maior contingente de pessoas em situação de prostituição, resultando em níveis acentuados de vulnerabilidade^3^.

No presente estudo, as comorbidades e a falta de acesso à vacinação e ao uso de medidas protetivas, inseridos no contexto dessas mulheres, receberam destaque como agravantes do risco de infecção por SARS-CoV-2, corroborando com dados descritos na literatura recente^4^.

Dentre as 219 entrevistadas, 86 relataram apresentar comorbidades. As mais citadas foram hipertensão arterial (HA) (40,7%), pneumopatias crônicas graves (24,4%), imunossupressão (22,1%) e diabetes mellitus (19,8%). Segundo as Diretrizes Brasileiras de Hipertensão Arterial de 2020, 21,4% da população brasileira relata HA, prevalência que aumenta para 65% em pessoas acima dos 60 anos^5^. Neste estudo, entretanto, a média de idade das entrevistadas foi de 41,3 anos, sendo que apenas 7,3% apresentavam mais de 60 anos. A disparidade entre idades permite inferir que tais mulheres têm incidência aumentada de HA em idades mais precoces, possivelmente devido à exacerbação de outros fatores de risco.

De acordo com o Plano Nacional de Imunização (PNI), indivíduos que possuem comorbidades pertencem a um dos grupos prioritários na fila de vacinação contra a covid-19. Contudo, neste estudo, apenas 26 das 86 mulheres que relataram possuir doenças haviam se vacinado (30,2%). Dessa forma, mesmo que a prioridade aos enfermos seja considerada pelas instâncias governamentais, parece que essa informação não chega à população mais vulnerável. A vacinação das mulheres em situação de prostituição depende do seu acesso aos centros sociais e de saúde^1^. O estigma que paira sobre elas pode ser um dos fatores que as distâncias dos serviços públicos sociais e de saúde, entre eles as campanhas de imunização. Historicamente, essas mulheres foram e ainda são vistas apenas como “reservatórios das doenças”, transmissoras de IST, não levando em conta o risco de adoecimento que correm.

Em relação às máscaras, podemos perceber que a maioria das mulheres as utilizava (59,4%); todavia, essa proporção está aquém do satisfatório frente a situação sanitária da covid-19 e o modo de transmissão do vírus. Uma das explicações é que o número de clientes fixos diminuiu devido à pandemia, o que acarretou a aceitação de novos clientes, com os quais essas mulheres não possuem relação de confiança ou conhecimento acerca dos cuidados contra a covid-19^6^. Ademais, esses clientes podem exigir a retirada da máscara durante o encontro^6^. Semelhante ao combate ao HIV, o combate à covid-19 nessa situação é extremamente complexo^6^ e, apesar de medidas educacionais e da doação de máscaras para essas mulheres, muitas continuam realizando programas sem o uso de máscara por necessidade financeira. Dessa forma, a dificuldade no uso de máscara durante o programa deixa explícita a necessidade de cuidados específicos para essa população, como por exemplo, a prioridade na vacinação.

## CONCLUSÃO

O presente estudo indica que as mulheres em situação de prostituição entrevistadas foram, em sua maioria, negras, de meia idade, pobres e não vacinadas contra covid-19. Assim, além de articularem desigualdades de gênero, raça e classe, essas mulheres são expostas a um maior risco de contraírem covid-19, devido às condições de trabalho e ao maior risco de apresentarem doença grave, por causa da idade e da falta de vacinação. Demais aspectos, como prevalência de comorbidades, falta de uso de máscaras durante o programa e impossibilidade de distanciamento social também se somam a esses fatores, tornando o cenário ainda mais delicado.

Por fim, o impacto da falta de acesso à informação e aos serviços de saúde já disponíveis não deve ser menosprezado, uma vez que é um ponto de precariedade notável na atenção a esse público.

As múltiplas vulnerabilidades apresentadas, portanto, apontam a necessidade premente de políticas públicas específicas para essa população, localizada em uma área já apagada como é o centro da cidade de São Paulo. Atualmente, essas mulheres são invisíveis para a sociedade e para o Sistema Único de Saúde (SUS), quando não são respeitados seus princípios de universalidade, integralidade e equidade no acesso à saúde. Assim, a visibilidade das mulheres em situação de prostituição em todos os contextos, sobretudo no de pandemia, é crucial para que a saúde pública no Brasil atinja seus princípios.

## Footnotes

a Ministério da Saúde (BR), Secretaria de Vigilância em Saúde, Departamento de Imunização e Doenças Transmissíveis, Coordenação-Geral do Programa Nacional de Imunizações. Plano Nacional de Operacionalização da Vacinação contra a COVID-19. 4a. ed. Brasília, DF; 15 de fevereiro de 2021. [citado 12 set 2021]. Disponível em: https://www.gov.br/saude/pt-br/media/pdf/2021/janeiro/29/PlanoVacinaoCovid_ed4_15fev21_cgpni_18h05.pdf

b Brazil: WHO Coronavirus Disease (COVID-19) dashboard with vaccination data. Geneva (CH): WHO; 2021 [citado 12 set 2021]. Disponível em: https://covid19.who.int/region/amro/country/br

c São Paulo (Estado). #Vacina Já. São Paulo; 2021 [citado 12 set 2021]. Disponível em: https://www.vacinaja.sp.gov.br/

d Secretaria Municipal de Saúde de São Paulo. Instrutivo n°21 para Priorização de Doses. Campanha Vacina Sampa contra a COVID-19. São Paulo; 2021 [citado 18 out 2021]. Disponível em: https://www.prefeitura.sp.gov.br/cidade/secretarias/saude/vigilancia_em_saude/doencas_e_agravos/coronavirus/index.php?p=312200
